# Internet-Based Interventions for Preventing Premature Birth Among Pregnant Women: Systematic Review

**DOI:** 10.2196/54788

**Published:** 2024-04-02

**Authors:** Sun-Hee Kim, Jin-Hwa Park, Sun-Young Jung, Jennie C De Gagne

**Affiliations:** 1 College of Nursing Research Institute of Nursing Science Daegu Catholic University Daegu Republic of Korea; 2 School of Nursing Duke University Durham, NC United States

**Keywords:** anxiety, body weight, depression, gestational diabetes mellitus, high-risk behavior, internet-based interventions, neonatal outcomes, pregnancy, premature birth, pregnancy outcomes, stress, systematic review

## Abstract

**Background:**

Premature birth rates have slightly increased globally, making its prevention critical for both short-term and long-term health outcomes. Various interventions have been developed in response to the multifaceted risk factors for premature birth, including internet-based programs. These programs offer accessibility and enhanced engagement; however, their overall efficacy in preventing premature births requires thorough evaluation.

**Objective:**

This systematic review aims to identify the study designs and assess the effectiveness of internet-based interventions in preventing premature birth among pregnant women.

**Methods:**

A comprehensive search of the MEDLINE, Embase, CINAHL, and Cochrane Library databases was conducted to identify randomized trials and quasi-experimental studies evaluating internet-based interventions for premature birth prevention in pregnant women. The search was inclusive, with no restrictions based on language or geographical location, allowing for a comprehensive global perspective. The time frame for the inclusion of studies extended until February 2023. The risk of bias (RoB) in each study was independently assessed by 3 authors forming pairs, using the revised Cochrane RoB tool (RoB 2) for randomized trials, as per the PRISMA (Preferred Reporting Items for Systematic Reviews and Meta-Analyses) guidelines. Owing to heterogeneity in populations, measurements, and interventions, a meta-analysis was not conducted.

**Results:**

This review included 26 articles, comprising 12 intention-to-treat and 14 per-protocol studies. The overall RoB was high in most intention-to-treat studies and of some concern in most per-protocol studies. The target populations varied, including nonspecific pregnant women, those with gestational diabetes mellitus (GDM) or those at risk of GDM, individuals with anxiety or depression, and those experiencing preterm labor. Psychosocial, physiological, and wellness health outcomes were evaluated. Internet-based interventions effectively reduced stress/distress in nonspecific pregnant women but not in those experiencing preterm labor. Their effectiveness in reducing anxiety and depression varied, with inconsistent results among different groups. In women with GDM or those at risk of GDM, interventions successfully controlled fasting plasma glucose and 2-hour postprandial plasma glucose levels but did not consistently manage glycated hemoglobin levels. These interventions did not reduce the incidence of premature births across the various populations studied. The effectiveness of these internet-based interventions in addressing substance or alcohol abuse and insomnia also varied.

**Conclusions:**

Internet-based interventions show promise in improving psychosocial health and managing blood sugar to prevent premature birth, highlighting variability in effectiveness across different risk factors. Further research, including clinical trials, is vital for developing, evaluating, and disseminating effective, safe internet-based interventions. Establishing standardized measurement tools and rigorous evaluation processes is crucial for enhancing these interventions’ effectiveness and reliability in clinical practice, significantly contributing to preventing premature births and improving maternal health outcomes.

**Trial Registration:**

PROSPERO CRD42021278847; https://www.crd.york.ac.uk/prospero/display_record.php?ID=CRD42021278847

## Introduction

### Background

The estimated global premature birth rate per 100 live births increased slightly from 9.8% (13.8 million premature births) in 2010 to 9.9% (13.4 million premature births) in 2020 [1]. This indicates an average annual increase rate in premature birth prevalence of 0.14% [1]. Complications of premature birth are the leading cause of childhood mortality, which refers to the probability of dying between birth and exactly 5 years of age, expressed per 1000 live births. This encompasses 35% of neonatal deaths and 18% of deaths in children aged <5 years [2]. Compared with their term-born peers, premature babies are more likely to develop respiratory distress syndrome, sleep apnea, necrotizing enterocolitis, and intraventricular hemorrhage in the neonatal period [3] and have worse cognitive, language, and motor development and social-emotional competence as children aged <5 years [4,5]. In addition, young adults born preterm are more susceptible to psychological fragility (in terms of anxiety and insecurity) and tend to exhibit lower cognitive ability [6]. Therefore, reducing the incidence of premature births is of utmost importance for the short- and long-term health and development of children.

Sociodemographic, nutritional, medical, obstetric, and environmental factors can increase the risk of premature birth. Consequently, interventions have been developed to prevent or mitigate known modifiable risk factors for premature birth [7]. Primary and secondary prevention encompasses a wide range of interventions, including medication, surgical procedures, cervical devices, targeted diets, physical exercise, smoking cessation programs, nutritional supplementation, education, and various special tests or investigations [7,8]. For women to participate in preventive activities for primary health promotion; secondary activities; and tertiary activities, including lifestyle modification, health screening uptake, treatment compliance, and participation in rehabilitation programs, understanding and continuous motivation are required. Recently, internet-based interventions have been developed to provide health-related information to individuals who may not have direct access to medical facilities [9], offering accessibility and availability regardless of time and location [10]. In addition, internet-based interventions can enhance engagement with self-monitoring, promote health-related understanding, and increase knowledge and risk perception of disease as well as bolster self-efficacy in disease management [11].

Previous systematic reviews have thoroughly examined interventions aimed at preventing premature birth, evaluating the overall evidence for such interventions [7,8,12,13]. These reviews covered a range of specific interventions, including infection treatment [14], pharmacological methods [15-17], nutrient supplementation [18], cerclage [19], cervical devices [20], and social support [21]. However, to date, only 2 systematic reviews have explored internet-based interventions in this context, confirming the effectiveness of technology-supported lifestyle interventions [22] and telemedicine [23] specifically for pregnant women with gestational diabetes mellitus (GDM). Although the effectiveness of internet-based interventions for smoking cessation among pregnant women [24] and prenatal interventions for maternal health [25] has been established, their effectiveness in preventing premature birth remains unconfirmed.

### Objectives

To address this gap, we conducted a systematic review of randomized controlled trials (RCTs) and quasi-experimental studies focusing on internet-based interventions for premature birth prevention. The objectives of this review were three-fold: (1) to describe the general characteristics of the studies included, (2) to identify the study designs of internet-based interventions pertinent to premature birth prevention, and (3) to evaluate the effectiveness of internet-based interventions in achieving outcomes related to premature birth prevention among the target population.

## Methods

### Design

This systematic review was conducted in accordance with the PRISMA (Preferred Reporting Items for Systematic Reviews and Meta-Analyses) guidelines [26] and registered in PROSPERO (CRD42021278847). Our review focused on 2 specific research questions formulated using the Population, Intervention, Comparison, and Outcome strategy: (1) What is the efficacy of internet-based interventions in reducing the risk of premature birth among pregnant women compared to standard prenatal care? (2) How do internet-based interventions impact maternal health outcomes, such as stress, anxiety, and gestational diabetes, in pregnant women at risk of premature birth? These questions aimed to evaluate the effectiveness of internet-based interventions in both reducing premature births and improving crucial maternal health outcomes. Our systematic search targeted several electronic databases, including MEDLINE, Embase, CINAHL, and the Cochrane Library, focusing on studies published up to February 2023. To augment our database search, we manually reviewed the reference lists of the included publications.

### Eligibility Criteria

Our inclusion criteria encompassed published RCTs, quasi-experimental studies, and experimental studies on the prevention of premature birth. We imposed no restrictions regarding the country or language of publication. The target population included all pregnant women, including those with normal or high-risk pregnancies, pregnancy complications, or at risk of premature birth. The interventions were internet-based and used various devices, such as computers and mobile phones. We excluded cross-sectional, case-control, retrospective, and prospective cohort studies; noncomparator experimental studies; animal experiments; reviews; qualitative studies; case reports; unpublished data; and gray literature, such as conference abstracts, letters, editorials, dissertations, and unavailable full texts. Studies targeting prepregnant women, women in the postpartum period, women outside childbearing age, and men were also excluded.

### Search Strategy

Adapted search terms for each database included a combination of terms related to population (eg, “women”), pregnancy (eg, “premature birth” and “pregnancy”), information and communication technology (eg, “computer”), treatment (eg, “internet” and “online”), and study design (eg, “randomized controlled trial”). These terms were used to search titles, abstracts, keywords, or text words. The exact search terms are detailed in Multimedia Appendix 1.

### Selection and Data Collection Processes

All identified studies were first imported into a reference manager for deduplication. The titles and abstracts were then independently screened by 2 of the 3 reviewers, working in rotating pairs (ie, A and B, B and C, and A and C). Following this initial screening, relevant studies underwent a full-text review. Disagreements at this stage were resolved through discussion or consultation with the third reviewer, ensuring a consensus on the inclusion of studies. Any studies found to be irrelevant after full-text review were excluded from further consideration. Simultaneously, a data extraction form was collaboratively developed and pretested by the reviewers to systematically collect review characteristics and outcome data from the selected studies. This process of data extraction was also conducted independently by 2 pairs of reviewers. In cases of discrepancies in the extracted data, the reviewers engaged in discussions to reach a consensus or consulted the third reviewer for an objective resolution.

### Data Extraction

The extracted data included study characteristics (eg, authors, year, country of origin, research design, and sample size), study results (primary and secondary findings for outcome measures, including effect sizes), and intervention details (eg, name, method, timing, duration, and group type). Because of the variation in methodologies across studies, conducting a meta-analysis was considered unsuitable. Instead, information was synthesized narratively, categorizing outcomes into psychosocial, physiological, and wellness health outcomes. Effect sizes were calculated using means and SDs or frequencies and percentages depending on the study design.

### RoB Assessment

Two pairs of reviewers independently assessed the methodological quality using the revised Cochrane RoB tool for randomized trials [27]. This tool evaluates 5 domains: randomization process, deviation from intended interventions, missing outcome data, outcome measurement, and reported result selection grouped into 3 levels of RoB (low risk, some concern, and high risk). Studies were categorized into 2 groups: intention-to-treat (ITT) and per-protocol (PP), with disagreements resolved through discussion or consultation with a third person.

### Statistical Analysis

Owing to the heterogeneity in interventions and participant characteristics, we opted for a narrative synthesis instead of a meta-analysis. When available, effect sizes were calculated using data from the studies, using various metrics such as Cohen *d*, Cohen *f*, Morris *d*, Hedges *g*, Cohen *h*, odds ratio (*P* value and 95% CI), and relative risk (95% CI and *P* value) [28]. Of the 26 papers reviewed, 5 (19%) lacked sufficient statistical data to calculate the effect size of the intervention. We reached out to the authors of these papers for additional information. However, responses were not received for several of these inquiries, limiting our ability to calculate effect sizes for all studies. Consequently, effect sizes were calculated for 21 articles. In instances where additional data from the original authors were not obtained, our evaluations were based on the information available in the study. In cases where studies presented results solely in graphical form, we calculated effect sizes where possible, specifically if the graph provided measurable mean and SD. However, for graphics lacking detailed data, such as missing SDs, effect size calculation was not feasible.

## Results

### Overview

**Figure 1:**
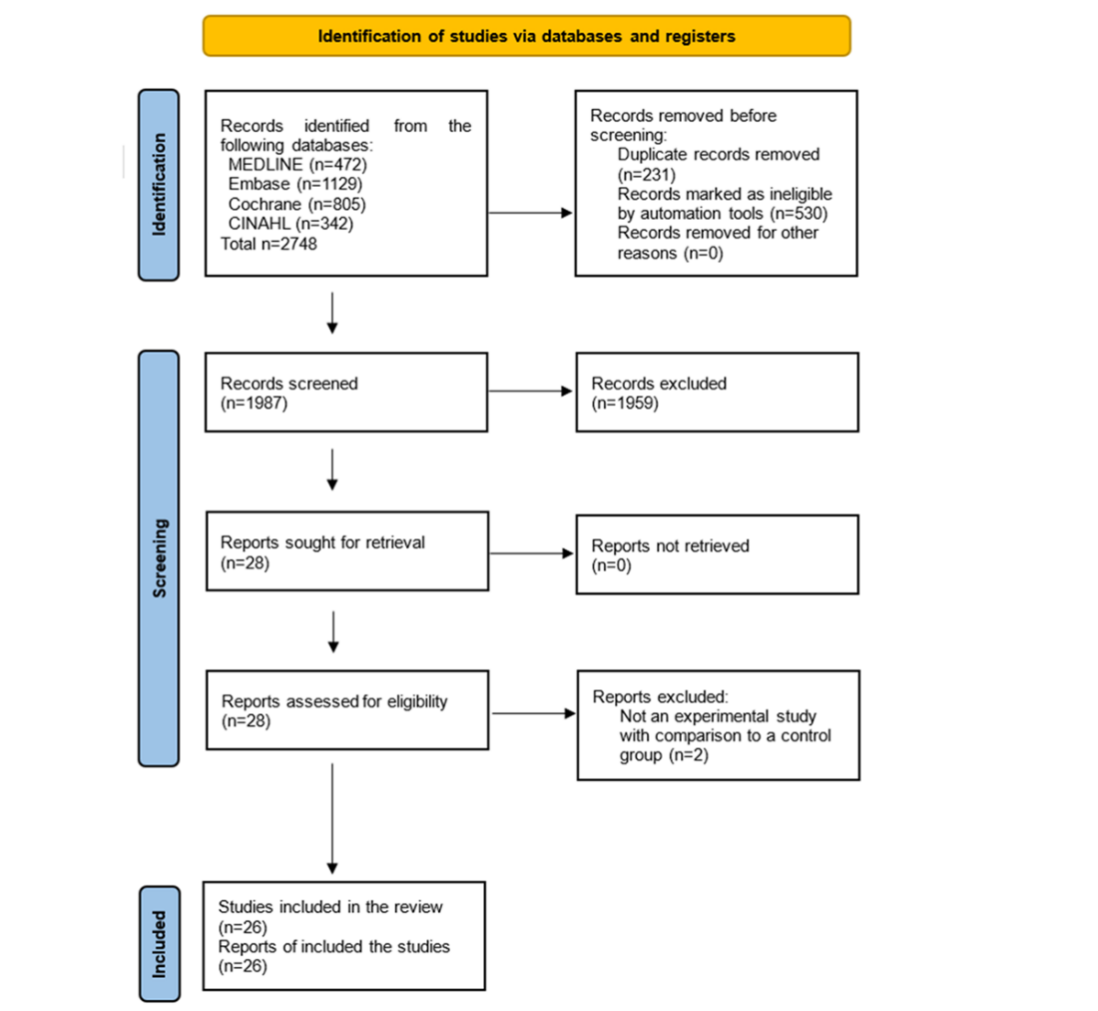
PRISMA (Preferred Reporting Items for Systematic Reviews and Meta-Analyses) diagram showing the selection of included studies.

Initially, a total of 2748 articles were retrieved from the 4 databases. After excluding 761 duplicate articles, 1987 remained. During the initial screening stage, 1959 papers were excluded after reviewing the study title and abstract. The full texts of the remaining 28 studies were reviewed, and 2 studies that were not controlled comparative experimental studies—they were 1-group pre- and postintervention comparison studies—were excluded. Finally, 26 studies were selected for the systematic review. Figure 1 illustrates the study selection process.

### Quality of the Studies and RoB Assessment

**Figure 2:**
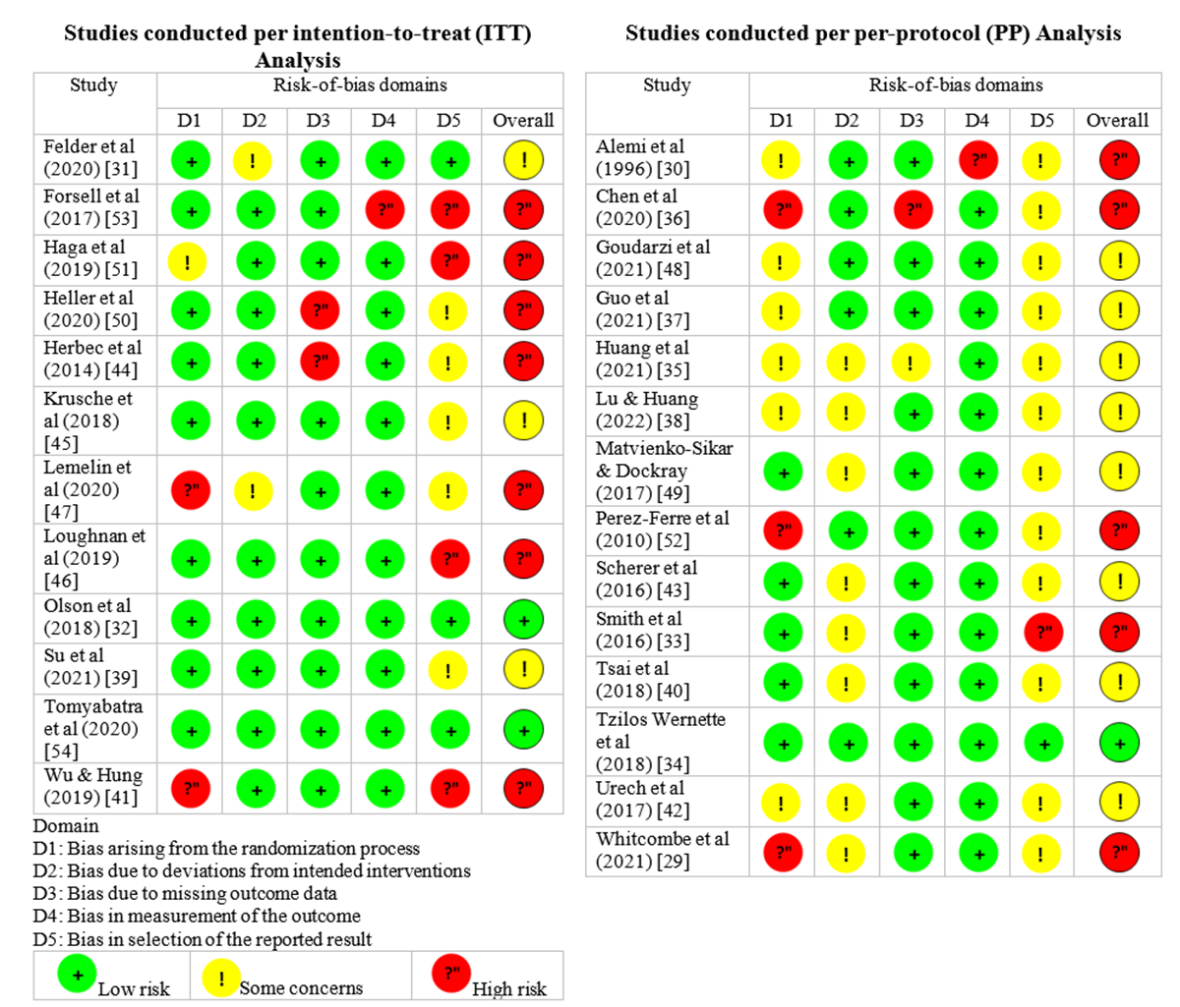
Risk-of-bias (RoB) assessment using the revised Cochrane RoB tool for randomized trials [29-54].

In the subset of studies that used ITT analysis, the overall RoB was categorized as low for 17% (2/12) of the studies, exhibiting some concerns in 25% (3/12) of the studies, and high in 58% (7/12) of the studies. Among the studies using PP analysis, only 7% (1/14) of the studies were assessed as having a low RoB, whereas 57% (8/14) of the studies had some concerns, and 36% (5/14) of the studies were deemed to have a high risk. The detailed outcomes of the RoB assessment for the 12 ITT and 14 PP studies are shown in Figure 2.

### Study Characteristics

The 26 studies included in this review were conducted across various countries, with the largest number from the United States (6/26, 23% studies [29-34]), followed by China (4/26, 15% studies [35-38]), Taiwan (3/26, 11% studies [39-41]), Switzerland (2/26, 8% studies [42,43]), and the United Kingdom (2/26, 8% studies [44,45]). In addition, 1 study each was conducted in Australia [46], Canada [47], Iran [48], Ireland [49], the Netherlands [50], Norway [51], Spain [52], Sweden [53], and Thailand [54]. The publication years spanned from 1996 to 2022, with most studies (14/26, 54%) [32-34,40-46,49,51-53] published between 2010 and 2019, 42% (11/26) of the studies [29,31,35-39,47,48,50,54] published from 2020 to the time of the review, and 4% (1/26) of the studies [30] published before 2000. Among these, one study was published in Chinese [35] and another in Persian [48], with the remaining studies all written in English.

With regard to research design, 81% (21/26) of the studies used a randomized controlled experimental design [30-39,42-46,49-54], whereas 19% (5/26) of the studies used a quasi-experimental design [29,40,41,47,48]. The focus of these studies varied, with 8% (2/26) of the studies targeting pregnant women diagnosed with anxiety or depression [46,53], 27% (7/26) focusing on pregnant women with GDM or at risk of GDM [29,35,37-39,47,52], and 8% (2/26) involving women experiencing preterm labor [42,43]. Of the 26 studies, 1 (4%) was dedicated to pregnant smokers [44], 2 (8%) were dedicated to pregnant women using drugs or alcohol [30,34], and 1 (4%) was dedicated to those experiencing insomnia [31]. The remaining 42% (11/26) of the studies targeted nonspecific pregnant populations [32,33,36,40,41,45,48-51,54]. Detailed information on these studies is provided in Tables 1 and 2.

**Table 1:** Overview of the general characteristics of the studies (N=26).

Characteristics and category		Values, n (%)
**Country**		
	Australia [46]	1 (4)
	Canada [47]	1 (4)
	China [31,37-39]	4 (15)
	Iran [48]	1 (4)
	Ireland [49]	1 (4)
	Netherlands [50]	1 (4)
	Norway [51]	1 (4)
	Spain [52]	1 (4)
	Sweden [53]	1 (4)
	Switzerland [29,43]	2 (8)
	Taiwan [40-42]	3 (11)
	Thailand [54]	1 (4)
	United Kingdom [44,45]	2 (8)
	United States [30,32-36]	6 (23)
**Publication year**		
	<2000 [32]	1 (4)
	2000 to <2010	0 (0)
	2010 to <2020 [29,34-36,41-46,49,51-53]	14 (54)
	>2020 [30,31,33,37-40,47,48,50,54]	11 (42)
**Publication language**		
	Chinese [35]	1 (4)
	Persian [48]	1 (4)
	English [29-34,36-47,49-54]	24 (92)
**Research design**		
	RCT^a^ [29,31-40,43-46,49-54]	21 (81)
	Quasiexperimental trial [30,41,42,47,48]	5 (19)
**Participants’ characteristics**		
	Anxiety or depression [46,53]	2 (8)
	GDM^b^ or at risk of GDM [30,31,38-40,47,52]	7 (27)
	Preterm labor [29,43]	2 (8)
	Smoking [44]	1 (4)
	Drug or alcohol use [32,36]	2 (8)
	Insomnia [33]	1 (4)
	Nonspecific [34,35,37,41,42,45,48-51,54]	11 (42)
**Intervention type**		
	Website [29,30,34,35,41,43-47,49-51,53]	14 (54)
	Computerizing intervention authoring system [36]	1 (4)
	Website or mobile app [33]	1 (4)
	Social network service [42]	1 (4)
	Offline and instant messenger [38]	1 (4)
	IoT^c^ and instant messenger [39]	1 (4)
	Only instant messenger [31,37,40,48,54]	5 (19)
	Telemedicine system and SMS text messaging [52]	1 (4)
	Electronic voice bulletin board by mobile [32]	1 (4)
**Intervention delivery method**		
	Individual [29,30,33-36,38-41,43-47,49-53]	20 (77)
	Group [31,32,37,42,48,54]	6 (23)
**Intervention timing of pregnancy period**		
	≤First trimester	0 (0)
	≤Second trimester [49]	1 (4)
	≤Third trimester [29-39,41-48,50,52-54]	23 (89)
	≤Post partum [40,51]	2 (8)
**Intervention duration^d^**		
	Approximately 1 to 4 weeks [36,45,46,49]	4 (15)
	Approximately 4 weeks and 1 day to 8 weeks [29,33,43,44,48,50]	6 (23)
	Approximately 8 weeks and 1 day to 12 weeks [39,51,53]	3 (11)
	Approximately 12 weeks and 1 day to 16 weeks [32]	1 (4)
	Approximately 16 weeks and 1 day to 20 weeks [31,47,52]	3 (11)
	Approximately 20 weeks and 1 day to 24 weeks [40,41]	2 (8)
	Approximately 24 weeks and 1day to 28 weeks [35,38,42]	3 (11)
	Approximately 28 weeks and 1 day to 32 weeks [34]	1 (4)
	Approximately 32 weeks and 1 day to 36 weeks [54]	1 (4)
	Not reported [30,37]	2 (8)
**Comparator**		
	Usual antenatal care [31,39,40,42,51]	5 (19)
	Usual antenatal health education [37,41,54]	3 (11)
	Usual treatment [33,46,49]	3 (11)
	Watching brief segments of popular television shows with subsequent questions [36]	1 (4)
	One-page static, nonpersonalized website that provided brief standard advice [44]	1 (4)
	None [29,30,32,34,35,38,43,45,47,50,52,53]	12 (46)
	Not reported [48]	1 (4)

^a^RCT: randomized controlled trial.

^b^GDM: gestational diabetes mellitus.

^c^IoT: Internet of Things.

^d^Categorized based on maximum duration.

**Table 2:** Summary of the study designs for internet-based interventions in pregnant women (N=26).

Study, year; country	Study design (analysis sets)	Participants (intervention n/control n)	Experimental intervention	Intervention method and group type (I^a^ or G^b^)	Intervention timing and duration	Comparative intervention
Alemi et al [30], 1996; United States	2-armed RCT^c^ (PP^d^)	Pregnant drug-using participants from the previous study (28/25)	Talknet: a voice bulletin board for electronic self-help and group support	Communication via electronic bulletin boards: touch tone telephone (G)	Third trimester, intrapartum and 4 months	None
Chen et al [36], 2020; China	2-armed RCT (PP)	Pregnant women (83/85)	Health education on enhancing the compliance	Instant messenger by mobile phone	First, second, and third trimester and NR^e^ (≥3 web-based courses)	Usual antenatal health education
Felder et al [31], 2020; United States	2-armed RCT (ITT^f^)	<28 weeks’ gestation with insomnia (105/103)	Sleepio (Big Health), digital cognitive behavioral therapy for insomnia	Website or mobile app (I)	First, second, and early third trimester (≤GA^g^ 32 wks) and 6 wks	Usual treatment
Forsell et al [53], 2017; Sweden	2-armed RCT (PP)	10-28 weeks’ gestation with depression (22/20)	ICBT^h^ for antenatal depression	Website (I)	Late first, second, and third trimester and 10 wks	None
Goudarzi et al [48], 2021; Iran	2-armed quasi-experimental trial (PP)	<24 weeks’ gestation (12/13)	Web-based unified transdiagnostic treatment on mental health problems	Instant messenger by mobile phone (G)	First, second, and early third trimester and 8 wks	NR
Guo et al [37], 2021; China	2-armed RCT (PP)	24-28 weeks’ gestation; first-diagnosed GDM^i^ (70/70)	Online-offline integrated medical care management	Offline and instant messenger by mobile phone (I)	Late second and third trimester and approximately 24-28 wks	None
Haga et al [51], 2019; Norway	2-armed RCT (ITT)	21-25 weeks’ gestation (678/664)	Mamma Mia: a universal preventive intervention for perinatal depressive symptoms	Website (I)	Second and third trimester as well as post partum and 11.5 mo	Usual perinatal care
Heller et al [50], 2020; The Netherlands	2-armed RCT (ITT and PP)	<30 weeks’ gestation (79/80)	MamaKits Online (internet-based problem-solving treatment) of depression and anxiety in pregnancy	Website (I)	First, second, and third trimester and 5 wks	None
Herbec et al [44], 2014; United Kingdom	2-armed RCT (ITT)	Pregnant smoking women (99/101)	MumsQuit: internet-based smoking cessation	Internet-based, access face-to-face, or telephone support (I)	First, second, and third trimester and 4 wks of prequit date support and up to 4 weeks of postquit date support (8 weeks)	One-page static, nonpersonalized website that provided brief standard advice
Huang et al [35], 2021; China	2-armed RCT (PP)	Pregnant women with GDM (144/151)	Mobile health management on GDM	Instant messenger by mobile phone (G)	Late second and third trimester and approximately 12-20 wks	Usual prenatal care
Krusche et al [45], 2018; United Kingdom	2-armed RCT (ITT)	12-34 weeks’ gestation (107/78)	Be Mindful online	Website (I)	Late first, second, and third trimester and 4 wks	None
Lemelin et al [47], 2020; Canada	2-armed quasi-experimental trial (ITT)	21-30 weeks’ gestation with newly diagnosed GDM (80/81)	Telehomecare (THCa) program for GDM management	Website (I)	Late second and third trimester and approximately 10-20 wks (until delivery)	None
Loughnan et al [46], 2019; Australia	2-armed RCT (ITT)	13-30 weeks’ gestation with anxiety or depression (18/33)	MUMentum (unguided ICBT) for antenatal anxiety and depression	Website (I)	Second and third trimester and 4 wks	Usual treatment
Lu and Huang [38], 2022; China	2-armed RCT (PP)	Pregnant women with GDM (44/44)	Fetal monitoring using Internet of Things and GDM educational information	Instant messenger by mobile phone (I)	Late second and third trimester and 3 mo	Usual prenatal care
Matvienko-Sikar and Dockray [49], 2017; Ireland	3-armed RCT; 2-armed RCT for analysis (PP)	10-22 weeks’ gestation (24/12)	Online mindfulness and gratitude intervention (body scan and reflection intervention)	Websites (I)	Second trimester and 3 wks	Usual treatment
Olson et al [32], 2018; United States	2-armed RCT (ITT and PP)	≤20 weeks’ gestation (1126/563)	e-Mom, m-Mom: self-help, integrated mobile phone and web-based behavior change intervention in preventing excessive gestational weight gain	Website (I)	Late first, second, and third trimester and 28-30 wks	None
Perez-Ferre et al [52], 2010; Spain	2-armed RCT (PP)	24-28 weeks’ gestation with GDM (49/48)	Telemedicine system	Telemedicine system based on internet and SMS text messaging (I)	Late second and third trimester and approximately 16-18 wks	None
Scherer et al [43], 2016; Switzerland	2-armed RCT (PP)	18-32 weeks’ gestation with preterm labor (31/27)	Internet-based cognitive behavioral stress management	Website (I)	Late second and third trimester an 6 wks	None
Smith et al [33], 2016; United States	2-armed RCT (PP)	10-14 weeks’ gestation (24/21)	Web-based behavioral intervention preventing excessive gestational weight gain	Website (I)	Late first, second, and third trimester, (3 weeks during GA approximately 10-36 wks) and approximately 20-26 wks	None
Su et al [39], 2021; Taiwan	2-armed RCT (ITT)	Pregnant women with GDM risk factors (56/56)	Web-based health management in preventing women at high risk of GDM from developing metabolic syndrome	Website, instant messenger by mobile phone (I)	Late second and third trimester as well as approximately 6-12 wks post partum and 6 mo	Usual prenatal care
Tomyabatra [54], 2020; Thailand	2-armed RCT (ITT)	Pregnant women (602/558)	Antenatal health education using audio-video social network about severe obstetric symptoms	Instant messenger: LINE app by mobile phone (G)	First, second, and third trimester and approximately 30-36 wks	Usual antenatal health education
Tsai et al [40], 2018; Taiwan	2-armed quasi-experimental trial (PP)	16-24 weeks’ gestation (68/67)	Web-based antenatal care system and routine antenatal education	Website (I)	Second and third trimester and approximately 12-22 wks	Usual antenatal health education
Tzilos Wernette et al [34], 2018; United States	2-armed RCT (PP)	Unplanned pregnant women, condomless sex, and alcohol or drug use or at risk for prenatal alcohol/drug use (31/19)	Health Checkup for Expectant Moms of substance use and risky sex	Computerizing intervention authoring system (I)	First, second and third trimester and approximately 75 min	Watching brief segments of popular television shows with subsequent questions
Urech et al [42], 2017; Switzerland	2-armed RCT (PP)	18-32 weeks’ gestation diagnosed preterm labor (50/43)	Internet-based cognitive behavioral stress management	Website (I)	Late second and third trimester and 6 wks	None
Whitcombe et al [29], 2021; United States	3-armed quasi-experimental trial (ITT)	21-30 weeks’ gestation with newly diagnosed GDM (21/45/37)	Web-Based Instruction on Nutrition	Website (I)	Late second and third trimester and NR	None
Wu and Hung [41], 2019; Taiwan	2-armed quasi-experimental trial (ITT)	<12 week’s gestation (66/55)	The Expectant Mother Club: Virtual Community	Closed social network community: closed community (G)	First, second, and third trimester and at least 24-26 wks	Usual prenatal care

^a^I: individual.

^b^G: group.

^c^RCT: randomized controlled trial.

^d^PP: per-protocol.

^e^NR: not reported.

^f^ITT: intention-to-treat.

^g^GA: gestational age.

^h^ICBT: internet-delivered cognitive behavioral therapy.

^i^GDM: gestational diabetes mellitus.

### Intervention Characteristics

#### Targeted Health Issue

As shown in Table 2, the interventions in the reviewed studies varied widely. Of the 26 studies, 10 (38%) focused on mental health treatment: 2 (20%) studies focused on web-based mindfulness [45,49], 7 (70%) studies focused on various psychological treatments [42,43,46,48,50,51,53], and 1 (10%) study focused on insomnia [31]. Furthermore, 19% (5/26) of the studies examined antenatal care interventions: 1 (20%) for nonspecific pregnant women [40] and 4 (80%) for those with GDM [35,37,47,52]. Education was the focus of 11% (3/26) of the studies, with 1 (33%) study focusing on diabetes diet education [29] and 2 (33%) studies focusing on general pregnancy health education [36,54]. Five studies targeted health behavior interventions, including diabetes dietary intake and exercise management [38,39], prenatal weight management behavior [32,33], and health care for substance abuse and risky sexual behavior [34]. Three studies explored self-help groups, including a web-based community for nonspecific pregnant women [41], a group for pregnant women with substance abuse [30], and a support group for smoking cessation [44].

#### Intervention Method and Group Type

Overall, 54% (14/26) of the studies used websites for intervention delivery [29,32,33,40,42-47,49-51,53], whereas 19% (5/26) of the studies used instant messaging only [35,36,38,39,48,54]. Various other methods were used in individual studies, such as computerizing intervention authoring systems [34], a mix of websites or mobile apps [31], social network services [41], offline and instant messengers [37], Internet of Things and instant messengers [38], telemedicine systems and SMS text messaging [52], and electronic voice bulletin boards using mobile devices [30]. Six studies targeted groups [30,35,36,41,48,54], and the remaining 20 studies focused on individual interventions.

#### Intervention Timing, Duration, and Comparative Approaches

The timing and duration of the interventions varied across studies. Although no interventions were exclusively conducted during the first trimester, 4% (1/26) of the studies covered the first and second trimesters [49], 88% (23/26) of the studies spanned all trimesters [29-38,40-48,50,52-54], and 8% (2/26) of the studies extended into the postpartum period [39,51]. The duration ranged from ≤4 weeks in 15% (4/26) of the studies [34,45,46,49] to >32 weeks in 4% (1/26) of the studies [54], with 8% (2/26) of the studies not specifying the period [29,36]. With regard to comparative interventions, 50% (13/26) of the studies provided standard care or education to the control groups, such as usual antenatal care [35,38,39,41,51], health education [36,40,54], or typical treatments [31,46,49]. Unique approaches included watching brief television segments with questions [34] and accessing a basic, nonpersonalized website [44]. Of the 13 studies, 12 (92%) did not provide any intervention to control groups [29,30,32,33,37,42,43,45,47,50,52,53] and 1 (8%) did not report the intervention in the control group [48]. This variety in intervention timing, duration, and comparative approaches underscores the diversity in study methodologies and target populations (Table 2).

### Outcomes and Effects of Interventions

**Figure 3:**
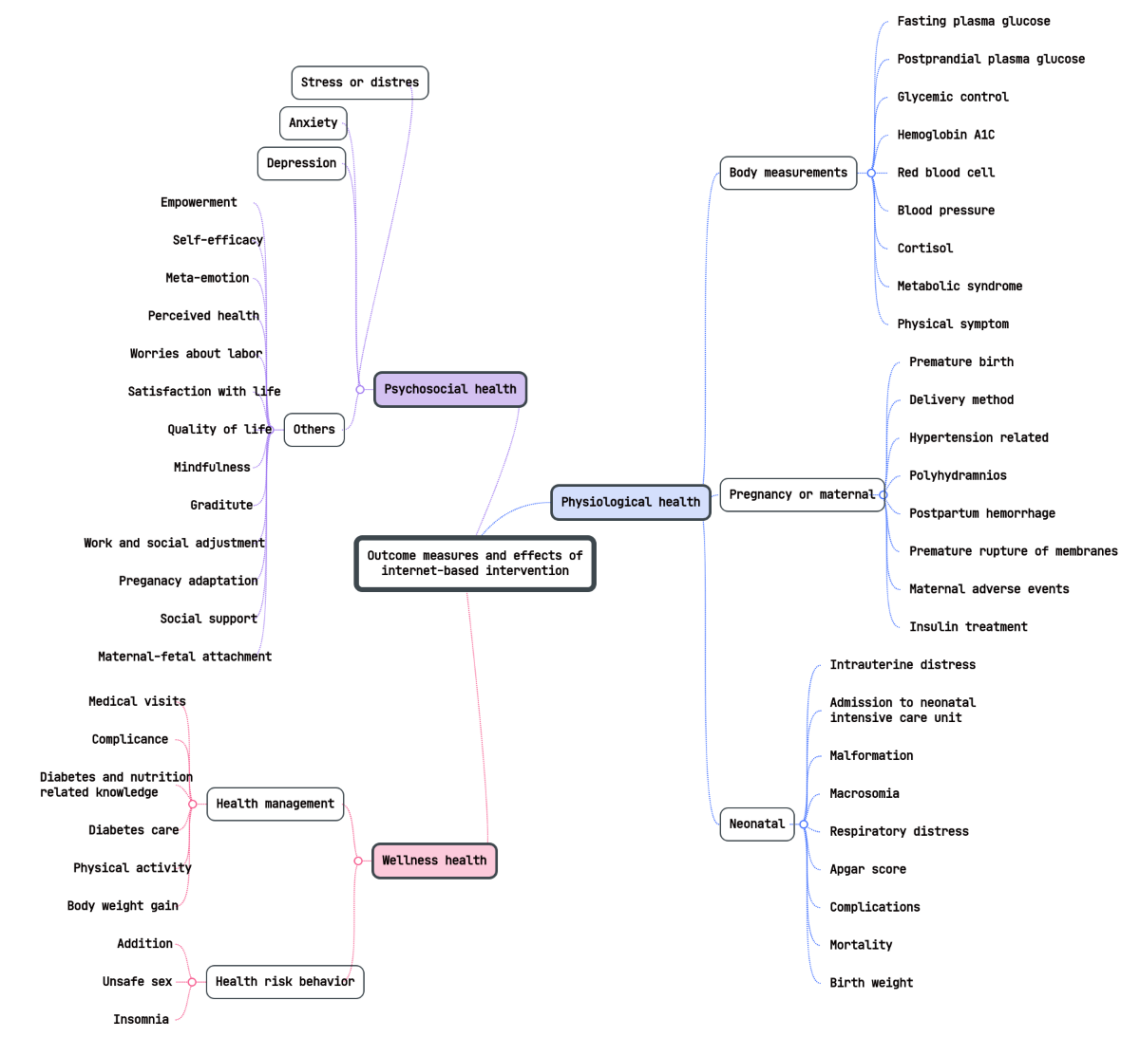
Mind map of the outcomes and health impacts of internet-based interventions. BP: blood pressure; NICU: neonatal intensive care unit; RBC: red blood cell.

The outcomes of the interventions, as detailed in Multimedia Appendix 2 [29-54], were divided into 3 main categories: psychosocial, physiological, and wellness health outcomes. Each category encompasses several subcategories, as shown in Figure 3.

#### Psychosocial Health Outcomes

Within this domain, a total of 16 distinct outcomes were identified. These psychosocial health outcomes encompassed a range of factors, including stress or distress, anxiety, and depression, along with a variety of other elements related to mental, emotional, and social well-being.

##### Stress or Distress

Seven studies assessed stress or distress, with 4 (57%) targeting nonspecific pregnant women [40,45,48,49], 1 (14%) focusing on women with anxiety or depression [46], and 2 (29%) on those with preterm labor [42,43]. Stress or distress was the primary outcome in 6 (86%) of these 7 studies [40,42,43,45,48,49]. Among the 7 studies, 2 (28%) specifically addressed populations with stress issues [42,43], whereas 2 (28%) other studies involved nonspecific pregnant women without preexisting stress issues [45,49]. Significant reductions in stress or distress were observed in the intervention groups of the studies involving nonspecific pregnant women [40,45,48,49]. In a study targeting women with anxiety or depression, a significant reduction in distress was noted 1 week after the intervention, but this effect was not sustained at the 4-week mark [46]. Conversely, interventions in studies involving women with preterm labor did not yield significant effects [42,43].

##### Anxiety

Of the 9 studies examining anxiety, significant reductions were observed under certain conditions. One study targeting women with GDM reported immediate reductions in anxiety after the intervention [37]. Another study, focusing on individuals with anxiety or depression, noted a significant reduction in anxiety 9 weeks after the intervention [46], and a study targeting those with insomnia observed significant reductions after 10 and 18 weeks [31]. However, no significant changes in anxiety levels were observed in 4 studies involving nonspecific pregnant women [45,48,50,53] or in 2 studies involving women experiencing preterm labor [42,43]. Notably, anxiety was the primary outcome in only 1 of these studies [46].

##### Depression

In the 10 studies addressing depression, 3 (30%) reported significant reductions: 1 involving nonspecific pregnant women [45], 1 involving women diagnosed with GDM [37], and 1 focusing on individuals with insomnia [31]. A study targeting women with depression [53] found mixed results, with a decrease in 1 of the 2 assessments for depression. However, the interventions did not significantly impact depression in 4 studies with nonspecific pregnant women [41,49-51], 1 study with women having anxiety or depression [46], and 1 study involving preterm labor [42]. Only 3 of these studies measured depression as their primary outcome [50,51,53], and 1 study did not report its results [48].

##### Other Psychosocial Health Outcomes

This category encompasses various aspects of psychosocial health. Significant improvements were noted in empowerment [48], self-efficacy following meditation [40], and metaemotion [48]. In 4 studies assessing perceived health, no significant effects of the interventions were observed in 1 study involving nonspecific pregnant women [53], 1 study with individuals experiencing anxiety or depression [46], and 1 study with drug users [30]; however, a study involving women with GDM showed significant improvement in concise health status [37]. Individual studies measured labor-related worries [45], life satisfaction [49], and quality of life [46] but found no significant impact from the interventions. Mindfulness increased significantly in 1 of the 3 studies [45,48,49] targeting nonspecific pregnant women. These interventions did not yield significant effects on gratitude [49], work and social adjustment [53], pregnancy adaptation [41], or social support [41]. In studies involving pregnant women with anxiety or depression [46] and nonspecific pregnant women [41], maternal antenatal and maternal-fetal attachments were not significantly influenced by the interventions, respectively.

#### Physiological Outcomes

This section summarizes the physiological outcomes across 3 categories: body measurement, pregnancy or maternal, and neonatal.

##### Body Measurement

Nine studies focused on body measurement outcomes. Fasting plasma glucose (PG) levels, assessed in 3 studies, showed significant intervention effects in 2 studies on women with GDM [37,38] but not in women at GDM risk [39]. The 2-hour postprandial PG levels showed significant intervention effects in 2 studies [37,38]. One study reported significant intervention impacts on average glycemic control rates across fasting PG, 1-hour PG, 2-hour PG, and nighttime PG levels [35]. Glycated hemoglobin (HbA_1c_) levels, measured in 2 studies [38,52], revealed significant intervention effects in 1 study [38]. One study noted significant intervention impacts on red blood cell and hemoglobin levels [38]. However, 2 studies found no significant intervention effects on systolic or diastolic blood pressure [39,52]. Waking and evening salivary cortisol levels showed significant changes [49], but the cortisol awakening reaction did not change significantly in a study targeting nonspecific pregnant women [49]. A study on women with preterm labor, however, showed significant effects [42]. Metabolic syndrome assessments in 1 study [39] revealed significant intervention effects on triglyceride and cholesterol levels, and metabolic syndrome changes, but not on high-density lipoprotein levels or waist circumference [39]. In addition, physical symptoms in nonspecific pregnant women showed no significant changes [41].

##### Pregnancy or Maternal

Eight studies reported on premature birth; no significant intervention effects were found, including 5 (63%) studies with women with GDM or GDM risk factors [35,37,39,47,52], 1 (13%) study on women with preterm labor [42], and 2 (25%) studies with nonspecific pregnant women [50,54]. In studies involving women with GDM, interventions showed no effect on assisted vaginal [47] or cesarean deliveries [35,39,47]. Three studies reported no impact on hypertension-related outcomes such as preeclampsia [39], gestational hypertension [47], or urine albumin-to-creatinine ratio [52]. There were also no significant effects on polyhydramnios [37], postpartum hemorrhage [35,37], or premature rupture of membranes [35,47]. However, 1 study reported a significant effect of interventions on maternal adverse events [38]. No significant effects were observed on the number of insulin-treated women in 1 study [52], but significant effects were noted on the total contact per insulin-treated woman (total hours) in another study [52].

##### Neonatal

Five studies involving women with GDM [35,37-39,54] reported on neonatal outcomes. Interventions showed no significant effects on intrauterine distress [37], admission to neonatal intensive care [35,39], malformations [35], macrosomia [35,37], or respiratory distress (neonatal asphyxia) in 2 studies [35,37], although 1 study reported a significant effect [54]. Significant effects were observed on the Apgar score in one study [38] and on the number of neonatal complications in another study [38]. The effect of interventions on low birth weight was not significant in one study [35], but the effect was significant in another study [39]. Two studies reported significant effects on birth weight [38,39].

#### Wellness Health Outcomes

This section describes 2 categories of wellness outcome measures used in the studies: health management and health risk behavior.

##### Health Management

The domain of health management included 6 outcomes. One study focusing on women with GDM reported a significant increase in the frequency of medical visits [47]. In another instance, the use of WeChat for health education and schedule reminders led to an increase in prenatal examinations [36]. Notable improvements in adherence to dietary standards were observed in a separate study [38], although another study found no significant impact of diabetes and nutrition-related knowledge [29]. The effectiveness of internet-based interventions was also evident in a study that focused on the diabetes care profile, demonstrating significant positive outcomes [37]. Furthermore, a web-based intervention aimed at enhancing physical activity resulted in significant increases in 20- and 30-minute sessions of moderate to vigorous activity per week [33]. Body weight gain, an important metric in maternal health, was assessed in 4 studies [32,33,39,52]. One study found no significant effects on several metrics, including the percentage of women exceeding the upper limit of the total gestational weight gain (GWG) guidelines at 28 weeks of gestation, the rate of GWG from 32 weeks until delivery, and the total GWG during this period [32]. Meanwhile, 2 other studies observed no significant changes in overall body weight [39,52], yet 1 study highlighted significantly lower BMI changes in the intervention group between 36 and 40 weeks of gestation and better weight recovery of 6 to 12 weeks post partum [39]. Another study, which found no significant effects on total GWG and the percentage gain according to the Institute of Medicine recommendations, reported significant improvements in adherence to these GWG guidelines [33].

##### Health Risk Behavior

Three outcomes were included under health risk behaviors. In the area of addiction, encompassing drug or alcohol use, one study found no significant differences between the intervention and control groups [30], whereas another study noted a significant difference over time [30]. A study on smoking cessation among pregnant women reported no significant effect on 4-week smoking abstinence [44]. Similarly, no significant differences were observed in a study examining condomless vaginal sex [34]. The severity of insomnia, a concern during pregnancy, showed inconsistent results in 2 studies [31,53]. While one study targeting pregnant women found no significant effect on insomnia severity [53], another study focusing on pregnant women with insomnia documented significant improvements in insomnia symptom severity, sleep efficiency, and sleep quality following the intervention [31].

## Discussion

### Principal Findings

This systematic review presents a comprehensive evaluation of web-based intervention studies focused on the prevention of premature birth in pregnant women. Various web-based interventions and diverse groups of pregnant women were included in the analysis; however, a significant gap was noted in studies that directly confirmed the effects of these interventions on premature birth. The measurement variables used to ascertain the direct effects of the interventions varied, and there were few well-designed interventional studies. These findings echo those of a meta-analysis of web-based educational interventions in 2022 [55]. Among the analyzed studies, only 12% (3/26) of the studies had an overall low RoB. Many of the included studies had moderate to high risk, primarily owing to nonblinding, adherence issues, and selection bias. This highlights the need for more high-quality intervention studies in this field. Blinding in internet-based intervention studies poses a challenge because of the active participation requirement and common attrition. Consequently, RCTs may not always unfold as intended. Thus, statistical analysis requires adjustment for participant bias, and participant characteristics should be carefully considered during interpretation.

This study found that most web-based interventions primarily used websites, followed by mobile instant messaging, aligning with common methods used in web-based health education interventions [56]. Only 1 intervention in this review used a mobile app [31], although it predominantly relied on mobile instant messengers for information delivery and encouraging participation. This approach is prevalent in countries such as China, Taiwan, Thailand, and Iran [35-39,48,54]. Recent studies, including 1 study using WhatsApp with a chatbot for health promotion messages, have shown higher uptake rates for interventions [57]. Mobile apps, being more accessible than websites, have shown greater effectiveness for glycemic control [11]. Further studies should thus compare the efficacy of websites and mobile apps and explore the use of mobile instant messengers and apps in more depth.

Premature births have many unexplained causes, with preeclampsia, eclampsia, intrauterine growth restriction, spontaneous preterm labor, and preterm premature rupture of membranes being the common causes [58]. Nonspecific pregnant women were the most common target group in the reviewed web-based interventions, followed by pregnant women with GDM, preterm labor, substance use, depression, and anxiety. However, experimental research on women with specific health issues is lacking. Although 7 experimental studies focused on pregnant women with GDM, highlighting the importance of blood glucose (BG) self-management, none targeted women with hypertensive disorders, which is crucial for early detection and management. Furthermore, despite spontaneous premature labor and preterm premature rupture of membranes being leading causes of premature birth [58], only 2 experimental studies [42,43] focused on these conditions. Hence, there is a critical need for further experimental studies targeting pregnant women with specific health conditions.

In most of the reviewed studies, interventions spanned the entire duration of pregnancy, likely because of the increasing risk of premature birth as pregnancy progresses. These web-based interventions typically lasted for 4 to 8 weeks, with a few studies using longer durations. This contrasts with web-based interventions for nonpregnant adults, where longer durations are more common [59]. Given the evolving physical conditions during pregnancy, there is a growing need for long-term interventions that cover the entire pregnancy period, aligning with the emphasis on a holistic approach to pregnancy health care [60]. This approach encompasses not only the pregnancy period itself but also the early and prepregnancy stages as well as the postpartum period. Web-based interventions offer the advantage of accessibility and flexibility, making them suitable for long-term implementation compared to in-person interventions. However, the conclusive impact of the intervention duration remains to be determined, indicating the need for further research.

Our research found that the interventions had varying effects on stress or distress among different groups of pregnant women. Studies involving nonspecific pregnant women [40,45,48,49] and those focusing on women diagnosed with anxiety or depression [46] reported significant reductions in stress or distress following the interventions. This success can partly be attributed to the interventions being tailored to the specific needs of these groups. For instance, the study by Loughnan et al [46], which applied self-guided cognitive behavioral therapy, was specifically designed to target anxiety and depression, closely aligning with participants’ conditions. In contrast, interventions aimed at pregnant women with preterm labor did not achieve a similar reduction in stress or distress [42,43]. This disparity may be owing to the interventions in these studies, which were led by Scherer et al [43] and Urech et al [42], not being adequately customized to meet the unique needs of patients experiencing premature labor. Given that the condition of premature birth was not directly addressed [42], it is possible that the programs were less effective for these participants. In addition, as gestational age increased and fetal maturation progressed, the health risks for the newborn decreased [42], which might have influenced the perceived stress levels and the efficacy of the interventions.

The studies included in this research used various interventions, such as cognitive behavioral management [42,43,45,46,49], unified transdiagnostic treatment [48], and antenatal care system [40]. Each of these approaches has its own theoretical basis and methodological implications, which could affect the outcomes. Furthermore, the studies used different stress assessment tools, including the Perceived Stress Scale, Pregnancy Stress Rating Scale-36, and Prenatal Distress Scale. It is important to note that these are self-report screening questionnaires and not diagnostic assessment tools evaluated by trained professionals. This raises questions about the generalizability and applicability of the findings to individuals clinically diagnosed with stress or distress.

The interventions showed significant effects on anxiety in studies targeting women with anxiety [46], GDM [37], and insomnia [31]. However, no significant effects were noted in studies targeting women with preterm labor [42,43]. This aligns with the earlier findings regarding stress. The diversity of the interventions and measurement tools used across the studies, particularly as only 2 studies used the same tools [45,53], indicates the need for further research to reliably estimate these effects on anxiety. In addition, concerning depression, the cognitive behavioral interventions did not significantly impact pregnant women with anxiety or depression [46] or those with preterm labor [42] assessed by the Edinburgh Postnatal Depression Scale (EPDS). Similarly, women with depression [53] showed no significant changes when evaluated using the EPDS, though different results emerged from the Montgomery-Åsberg Depression Rating Scale. Furthermore, 2 studies targeting depression in general pregnant populations [50,51,53] used the EPDS but reported no significant effects [50,51]. The EPDS, although effective in detecting postpartum depression, might be less sensitive in identifying severe or prenatal depression [53]. Notably, in women with insomnia [31], depression significantly decreased when assessed with the EPDS and treated with cognitive behavioral methods. This raises questions about the validity of the EPDS during pregnancy, especially for those at a high risk for depression. Furthermore, most internet-based interventions for depression in pregnant women did not yield effective outcomes as measured by the EPDS. Ashford et al [61], however, claimed its effectiveness in perinatal depression. This discrepancy could be owing to differing methodological qualities; the studies in this review had a high overall RoB, whereas Ashford et al [61] included studies with average to high methodological quality.

This review underscores a notable inconsistency in the types of interventions, target audiences, and assessment tools used across the studies examined. Dennis [62] emphasized the importance of preventive interventions for mental health issues in pregnant women, particularly advocating for targeting women with identified risk factors [63]. In our analysis, the limited effects of interventions aimed at reducing anxiety or depression among nonspecific pregnant women [50,51] could be attributed to the absence of specific risk factors for these conditions. This observation suggests that the effectiveness of preventive psychosocial health interventions may be enhanced by tailoring them to the needs of women with identifiable risk factors.

Most studies in our review focused on verifying the effectiveness of BG control in women with GDM. The research demonstrated significant effects on both fasting BG and 2-hour BG levels in several studies [11,22,23,64], aligning with the findings from previous meta-analyses. However, variations were observed, such as in 1 study using a telemedicine system where HbA_1c_ levels were significantly lower in the experimental group [65]. Conversely, a study that provided educational information on GDM did not show a significant impact. These discrepancies might be because of factors such as the duration of the interventions, sensitivity of the indicators used, and the limited number of trials conducted. The physiological changes during pregnancy, such as iron deficiency and reduced life span of red blood cells, can affect the sensitivity of HbA_1c_ assays [65]. In contrast, glycated albumin, with a shorter half-life, may offer a more accurate measure of short-term glucose fluctuations. However, research on glycated albumin in pregnant women is still limited [11], suggesting an area that warrants further investigation in the future.

In terms of preventing premature birth, interventions targeting women with GDM or those at risk for GDM did not demonstrate a significant effect on this outcome [35,37,39,42,47,50,52,54]. These studies primarily assessed the role of internet-based intervention in addressing the causes of premature birth, analogous to managing diabetes for blood sugar control. The complexity of factors influencing premature birth makes it challenging to ascertain the direct preventive effects of these interventions. Hence, premature birth was not the primary outcome in any of the reviewed studies. Given the insufficient number of studies specifically examining each intervention type, it becomes necessary to continue evaluating premature birth as a secondary outcome in future research. This approach will further our understanding of how interventions can mitigate or eliminate the causes of premature birth, thereby enhancing maternal and neonatal outcomes.

### Strengths and Limitations

This systematic review contributes to the literature by methodically analyzing internet-based interventions aimed at preventing premature birth. Its main strength lies in its extensive focus on a broad spectrum of outcomes, covering a wide range of maternal and neonatal health aspects. Another key strength is the inclusivity of the review, encompassing a diverse range of populations. The absence of geographical or language restrictions in the selection criteria enhances the comprehensiveness and global applicability of the review. This broad and inclusive approach not only illuminates the potential of internet-based interventions in the pregnancy context but also delineates their limitations, thereby laying a foundation for future research endeavors and informing clinical practice with a more global perspective.

However, the review has several limitations that need to be considered. Its reliance on studies from only 4 databases may have missed relevant research from other sources, potentially limiting the scope of the findings. The exclusion of specific types of publications, such as conference abstracts and dissertations, coupled with limited responses from original authors for additional data, could have introduced publication bias. In addition, 2 studies translated from non-English language using Google Translator may have inaccuracies in translation, affecting the interpretation of these studies. Although backward and forward citation tracking of the final included articles was initially planned, it was not conducted because of resource constraints and the comprehensive nature of the initial search. The underrepresentation of studies with a low RoB in this review suggests the need for caution in generalizing the results. Moreover, the focus on studies predominantly from countries with high internet use may limit the applicability of the findings to regions with different internet access levels and use patterns. Therefore, the results should be interpreted with an understanding of these contextual differences.

### Conclusions

This systematic review uncovered a wide array of internet-based interventions that target risk factors associated with premature birth, with notable efficacy in the realms of psychosocial health and blood sugar management. However, interventions addressing other risk factors have shown a considerable diversity in measurement methods and a range of experimental effects. This variability points to an ongoing challenge in accumulating robust evidence. These findings underscore the critical necessity for future clinical trials to not only develop and test but also widely disseminate internet-based interventions that are both safe and effective. In addition, there is a pressing need for the creation of standardized measurement tools. Rigorous evaluation processes should be established to enhance the effectiveness and reliability of these interventions in clinical settings. Such efforts are essential for ensuring that these digital health solutions can effectively contribute to the prevention of premature births and improve maternal health outcomes on a broader scale.

## Appendix

Multimedia Appendix 1 Search strategy for the specific database.

Multimedia Appendix 2 Outcome measures and effects of internet-based intervention on pregnant women.

Multimedia Appendix 3 PRISMA 2020 Checklist.

## Abbreviations

BG: blood glucose
EPDS: Edinburgh Postnatal Depression Scale
GDM: gestational diabetes mellitus
GWG: gestational weight gain
HbA1c: glycated hemoglobin
ITT: intention-to-treat
PG: plasma glucose
PP: per-protocol
PRISMA: Preferred Reporting Items for Systematic Reviews and Meta-Analyses
RCT: randomized controlled trial
RoB: risk of bias
