# Supplementary material for: Internet-Based Interventions for Preventing Premature Birth Among Pregnant Women: Systematic Review
Source: JMIR Pediatr Parent. 2024 Apr 2;7:e54788. doi: 10.2196/54788 (PMC11022135; doi:10.2196/54788)
Supplement: Multimedia Appendix 1 [file pediatrics_v7i1e54788_app1.pdf]

Appendix 1. Search strategy for the specific database

Databases	No	Search term	Searched articles
Ovid MEDLINE (1946 - Feb 28, 2023)	1	Wom?n.tw. OR female.tw. OR women/	2,069,744
	2	prematur$.tw. OR preterm.tw. OR Premature Birth/ OR Obstetric Labor, Premature/ OR PTB.tw. OR (pregnanc$ adj1 (high-risk OR problem$ OR complicat$)).tw. OR Pregnancy, High-Risk/ OR Pregnancy Complications/	347,296
	3	(video OR $computer OR television OR virtual OR VR OR animation OR ((command-line OR graphic user) adj1 interface)).tw. OR Virtual Reality Exposure Therapy/ OR User-Computer Interface/	475,105
	4	((distance OR online OR on-line OR electronic OR web-based OR internet OR correspondence) AND (education OR learning OR $schooling OR courses OR intervention OR program)).tw. OR Education, Distance/ OR Computer-Assisted Instruction/ OR Internet/	223,047
	5	((randomized controlled trial OR controlled clinical trial).pt. OR randomized.ab. OR randomised.ab. OR placebo.ab. OR drug therapy.fs. OR randomly.ab. OR trial.ab. OR groups.ab. OR experimental.tw.) NOT (exp animals/ NOT humans.sh.)	5,594,548
	6	1 AND 2 AND (3 OR 4) AND 5	472
Ovid EMBASE (1974 - Feb 28, 2023)	1	Wom?n.tw. OR female.tw. OR female/	11,645,442
	2	prematur$.tw. OR prematurity/ OR preterm.tw. OR premature labor/ OR PTB.tw. OR (pregnanc$ adj1 (high-risk OR problem$ OR complicat$)).tw. OR high risk pregnancy/	396,887
	3	(video OR $computer OR television OR virtual OR VR OR animation OR ((command-line OR graphic user) adj1 interface)).tw. OR virtual reality exposure therapy/ OR virtual reality/ OR computer interface/	617,035
	4	((distance OR online OR on-line OR electronic OR web-based OR internet OR correspondence) AND (education OR learning OR $schooling OR courses OR intervention OR program)).tw. OR e-learning/ OR computer interface/ OR Internet/	348,962
	5	(Randomized controlled trial/ OR Controlled clinical study/ OR random$.ti,ab. OR randomization/ OR intermethod comparison/ OR placebo.ti,ab. OR (compare OR compared OR comparison).ti. OR ((evaluated OR evaluate OR evaluating OR assessed OR assess) and (compare OR compared OR comparing OR comparison)).ab. OR (open adj label).ti,ab. OR ((double OR single OR doubly OR singly) adj (blind OR blinded OR blindly)).ti,ab. OR double blind procedure/ OR parallel group$1.ti,ab. OR (crossover OR cross over).ti,ab. OR ((assign$ OR match OR matched OR allocation) adj5 (alternate OR group$1 OR intervention$1 OR patient$1 OR subject$1 OR participant$1)).ti,ab. OR experimental.ti, ab. OR (assigned OR allocated).ti,ab. OR (controlled adj7 (study OR design OR trial)).ti,ab. OR (volunteer OR volunteers).ti,ab. OR human experiment/ OR trial.ti.) not (((random$ adj sampl$ adj7 ("cross section$" OR questionnaire$1 OR survey$ OR database$1)).ti,ab. not (comparative study/ OR controlled study/ OR randomi?ed controlled.ti,ab. OR randomly assigned.ti,ab.)) OR (Cross-sectional study/ not (randomized controlled trial/ OR controlled clinical study/ OR controlled study/ OR randomi?ed controlled.ti,ab. OR control group$1.ti,ab.)) OR (((case adj control$) and random$) not randomi?ed controlled).ti,ab. OR (Systematic review not (trial OR study)).ti. OR (nonrandom$ not random$).ti,ab. OR "Random field$".ti,ab. OR (random cluster adj3 sampl$).ti,ab. OR ((review.ab. and review.pt.) not trial.ti.) OR ("we searched".ab. and (review.ti. OR review.pt.)) OR "update review".ab. OR (databases adj4 searched).ab. OR ((rat OR rats OR mouse OR mice OR swine OR porcine OR murine OR sheep OR lambs OR pigs OR piglets OR rabbit OR rabbits OR cat OR cats OR dog OR dogs OR cattle OR bovine OR monkey OR monkeys OR trout OR marmoset$1).ti. and animal experiment/) OR (Animal experiment/ not (human experiment/ OR human/)))	6,302,357
	6	1 AND 2 AND (3 OR 4) AND 5	1,129

Databases	No	Search term	Searched articles
Cochrane Central Register of Controlled Trials (1995 - Feb 28, 2023)	1	(Wom*n OR female):ti,ab,kw OR mh "Women"	929,595
	2	(prematur* OR preterm OR PTB OR (pregnanc*NEXT/1(high-risk OR problem$ OR complicat$))):ti,ab,kw OR mh "Premature Birth" OR mh "Pregnancy, High-Risk" OR mh "Obstetric Labor, Premature" OR mh "Pregnancy Complications"	65,394
	3	(video OR $computer OR television OR virtual OR VR OR animation OR ((command-line OR graphic user) adj1 interface)).tw. OR mh "Video Games" OR mh "Virtual Reality Exposure Therapy" OR mh "Virtual Reality" OR mh "User-Computer Interface"	10,536
	4	((distance OR online OR on-line OR electronic OR web-based OR internet OR correspondence) AND (education OR learning OR $schooling OR courses OR intervention OR program)).tw. OR mh "Education, Distance" OR mh "Internet"	10,739
	5	#3 OR #4	10,801
	6	"randomized controlled trial":pt OR "controlled clinical trial":pt OR "randomized":ti,ab,kw OR "randomly":ti,ab,kw OR "trial":ti,ab,kw OR "random":ti,ab,kw OR "RCT":ti,ab,kw OR "Randomization":ti,ab,kw OR "Quasi-Experimental":ti,ab,kw OR "Experimental Studies":ti,ab,kw	1,351,095
	7	#1 AND #2 AND #5 AND #6	805
EBSCO CINAHL (1993 - Feb 28, 2023)	1	Wom*n OR female	17,252,863
	2	(Premature* OR preterm) OR (Pregnanc* AND (high-risk OR problem* OR complicat*))	1,240,017
	3	(video OR *computer OR television OR virtual OR VR OR animation) OR ((command-line OR graphic user) AND interface) OR (((distance OR online OR on-line OR electronic OR web-based OR internet OR correspondence) AND (education OR learning OR *schooling OR courses OR intervention OR program))	12,181,930
	4	(AB("randomized controlled trial" OR "controlled clinical trial" OR "randomized" OR "randomly" OR "trial" OR "random" OR "RCT" OR "Randomization" OR "Quasi-Experimental")) OR "Experimental Studies"	5,413,222
	5	1 AND 2 AND 3 AND 4	539
