# Supplementary material for: Internet-Based Interventions for Preventing Premature Birth Among Pregnant Women: Systematic Review
Source: JMIR Pediatr Parent. 2024 Apr 2;7:e54788. doi: 10.2196/54788 (PMC11022135; doi:10.2196/54788)
Supplement: Multimedia Appendix 2 [file pediatrics_v7i1e54788_app2.pdf]

Appendix 2. Summary of outcome measures and effects of internet-based intervention on pregnant women

Outcomes	Outcome measures [References]	Effect size (between groups comparison) Post/FU	p-value (confidence interval) [a,b,c,d]
**Psychosocial Health**			
Stress or distress	Perceived Stress Scale (PSS) [48]	d=6.46	<.001(4.51, 8.42)[a]
	Kessler 10-item Psychological Distress scale [46]	Post/FU between groups<br>g=0.88/0.52	(0.34,1.43)[a]/(-0.07,1.10)[b]
	Prenatal Distress Scale [49]	Between groups<br>f=0.04	NA[a]
	Perceived Stress Scale [45]<br>Tilburg Pregnancy Distress Scale	Post/FU between groups<br>d=0.71/d=0.26<br>d=0.53/NA	.010(0.20, 0.20)[a]/.050(-0.35, 0.86)[b]<br>.050(0.02, 1.04)[a]/NR[c]
	Perceived Stress Scale [43]	d[e]=-0.17	.672[b]
	Pregnancy Stress Rating Scale-36 [40]	Between groups<br>d=0.62	<.001(-0.99, -0.28)[a]
	Perceived Stress Scale [42]	f= 0.86	NR[b]
Anxiety	Generalized Anxiety disorder Scale-7 [31]	Post/FU between groups<br>d=-0.42/d=-0.46	<.001(-0.26, -0.10)[a]/<.001(-0.18, -0.07)[a]
	General Anxiety Disorder-7 [53]	g=0.63	.100(-0.84, 2.10)[b]
	Van den Bergh Pregnancy Anxiety Scale [48]	NR	NR[c]
	Self-rating Anxiety scale [37]	Between groups: post time<br>d=-1.26	<.001(-1.63, -0.90)[a]
	Hospital Anxiety and Depression Scale-Anxiety subscale [50]	Post/FU1/FU2 between groups<br>d=0.01/d=0.01/d=-0.21	.95[b]/.98[b]/.36[b]
	General Anxiety Disorder-7 [45]	Post/FU between groups<br>d=0.39/NA (Not applicable?)	.050(-0.12, 0.89)[b]/NA[d]
	Generalized Anxiety Disorder 7-item [46]	Post/FU between groups<br>g=0.400/0.760	(-0.13,0.93)[b]/(0.17,1.35)[a]
	Spielberger State-Trait Anxiety Inventory [43]<br>Spielberger State-Trait Anxiety Inventory	d[e]=-0.06<br>d[e]=-0.27	.743[b]<br>.466[b]
	Pregnancy Related Anxiety Test (PRAT) [42]<br>Spielberger State-Trait Anxiety Inventory<br>Spielberger State-Trait Anxiety Inventory	f=0.93<br>f=0.91<br>f=3.82	NR[b]<br>NR[b]<br>NR[b]
Depression	Edinburgh postnatal depression Scale [31]	Post/FU between groups<br>d=-0.39/d=-0.48	<.001(-0.30, -0.11)[a]/<.001(-0.20, -0.07)[a]
	Edinburgh Postnatal Depression Scale [53]<br>Montgomery Åsberg Depression Rating scale	g=0.52<br>g=1.21	.31(-1.08, 2.12)[b]<br><.001(0.50, 2.92)[a]
	Self-rating Depression scale [37]	Between groups: post time<br>d=-1.45	<.001(-1.8179, -1.0737)[a]
	Edinburg Postnatal Depression Scale [48]	NR	NR[c]
	Edinburgh Postnatal Depression Scale [51]	Post/FU1/FU2 between groups<br>OR=0.74/OR=0.78/OR=0.72/OR=0.71	.082(0.52, 1.04)[b]/.191(0.55, 1.13)[b]/.141(0.46, 1.12)[b]/.149(0.45, 1.13)[b]

	Center for Epidemiological Studies Depression scale [50]<br>Edinburgh Postnatal Depression Scale	Post/FU1/FU2 between groups<br>d=-0.03/d=-0.02/d=-0.45<br>d=0.00/d=0.02/d=-0.23	 .890[b]/.940[b]/.060[b]<br>.100[b]/.910[b]/.260[b]
	Edinburgh Postnatal Depression Scale [45]	Post/FU between groups<br>d=0.86/NA	 .05(0.35, 1.38)[a]/NA[d]
	Edinburgh Postnatal Depression Scale [46]	Post/FU between groups<br>g=0.20/0.35	 (-0.32, 0.73)[b]/(-0.23, 0.93)[b]
	Edinburgh Postnatal Depression Scale [49]	Between groups:<br>f=0.06	 NA[b]
	Edinburgh Postnatal Depression Scale [42]	f=2.44	NR[b]
	Edinburgh Postnatal Depression Scale [41]	f=0.10	.40(-2.30, 0.92)[b]/.30(-2.61, 0.87)[b]
Other Psychosocial Health			
Empowerment	Kameda prenatal psychological empowerment scale [48]	d=4.32	<.001(2.89, 5.75)[a]
Self-efficacy	General Self-Efficacy scale [40]	Between groups<br>d=0.73	 <.001 (0.38, 1.08)[a]
Meta-emotion	Mitmansgruber meta-emotion scale [48]<br>positive dimension<br>negative dimension	 d=1.65<br>d=3.17	 <.001(0.74, 2.56)[a]<br><.001(1.99, 4.35)[a]
Perceived health	General health status [30]<br>Health perception<br>Physical functioning<br>Role functioning<br>Social functioning<br>Mental functioning<br>Pain level	Between groups, only post time<br>d=0.00<br>d=0.13<br>d=0.12<br>d=0.48<br>d=0.48<br>d=0.19	 ≥.05[b]<br>≥.05[b]<br>=.05[a]<br>≥.05[b]<br>≥.05[b]<br>≥.05[b]
	EQ-5D-3L (health and function) [53]	g=0.08	.800(-0.03, 0.20)[b]
	Concise Health Status Scale (SF-36) [37]	d=1.11	<.001(0.75, 1.46)[a]
	Patient Health Questionnaire 9-item scale [46]	Post/FU between groups<br>g=0.30/0.35	 (-0.24, 0.81)[b]/(-0.02, 0.93)[b]
Worries about labor	Oxford Worries about Labor Scale [45]	f=0.27	NA[b]
Satisfaction with life	Satisfaction with Life Scale [49]	Between groups<br>f=0.03	 NA[b]
Quality of life	World Health Organization Quality of Life scale [46]<br>Physical health<br>Psychological<br>Social<br>Environment	 g=-0.19/-0.13<br>g=-0.01/-0.14<br>g=0.28/0.26<br>g=0.07/NR	 (-0.71, 0.34)[b]/(-0.70, 0.45)[b]<br>(-0.54, 0.51)[b]/(-0.71, 0.44)[b]<br>(-0.25, 0.80)[b]/(-0.32, 0.84)[b]<br>(-0.45, 0.59)[b]/NR[c]
Mindfulness	Bauer Five Facet Mindfulness Questionnaire [48]	d=3.26	<.001(2.06, 4.45)[a]
	Mindfulness practice [45]	Post/FU between groups<br>NR/NR	 NR[c]/NR[c]
	Mindfulness Attention Awareness Scale [49]	Between groups:<br>f=0.07	 NA[b]
Gratitude	Gratitude during Pregnancy Scale [49]	Between groups:<br>f=0.04	 NA[b]
Work and social adjustment	Work and social adjustment scale-depression [53]<br>Work and social adjustment scale-pregnancy	g=-0.03<br>g=0.49	.700(-3.18, 3.12)[b]<br>.440(-2.27, 3.24)[b]

Pregnancy adaptation	Pregnancy Adaptation Scale [41]	f=0.02	.59(-5.32, 3.02)[b]/.750(-4.57, 3.31)[b]
Social support	Social Support Scale [41]	f=0.02	.92(-3.97, 3.62)[b]/.520(-2.61, 5.14)[b]
Maternal-fetal attachment	Maternal Antenatal Attachment Scale [46]	g=0.03/-0.16	(-0.49, 0.55)[b]/(-0.74, 0.41)[b]
	Maternal–Fetal Attachment Assessment Scale [41]	f=0.03	.53(-5.64, 10.97)[b]/.34(-4.70, 13.65)[b]
**Physiological**			
Body measurements			
Fasting plasma glucose	Fasting plasma glucose [37]	Between groups: post time<br>d=-1.77	 <.001(-2.16, -1.38)[a]
	Fasting blood glucose [38]	NA	NA[a]
	Fasting blood glucose [39]	f=0.07	NA[b]
Postprandial plasma glucose	2h postprandial plasma glucose (2hPG) [37]	Post time between groups<br>d=-0.71	 <.001(-1.06, -0.37)[a]
	Blood glucose 2 hours after the meal [38]	NA	NA[a]
Glycemic control	Average glycemic control rate [35]	h=0.14	.004[a]
HbA1c	HbA1c [38]	NA	NA[a]
	HbA1c [52]	f=0.06	>0.05[b]
RBC	RBC [38]<br>Hemoglobin	NA<br>NA	NA[b]<br>NA[b]
BP	BP [52]:<br>Systolic<br>Diastolic	 f=0.07<br>f=0.08	 >0.05[b]<br>>0.05[b]
	BP [39]:<br>Systolic<br>Diastolic	 f=0.11<br>f=0.12	 NA[b]<br>NA[b]
Cortisol	Salivary cortisol [49]<br>Waking cortisol<br>CAR<br>Evening cortisol	Between times: only intervention group<br>f=1.25<br>f=0.25<br>f=2.13	 .004[a]<br>.490[b]<br><.001[a]
	Salivary CAR [42]	Post/ FU Time group effect<br>0.20/ 0.22	 .006[a]/.003[a]
Metabolic syndrome	Triglyceride [39]<br>Cholesterol<br>High-density lipoprotein·<br>Waist circumference change<br>Metabolic syndrome change	f=0.14<br>f=0.16<br>f=0.04<br>d=0.28<br>NA	NA[a]<br>NA[a]<br>NA[b]<br>(-0.09, 0.65)[b]<br>NA[a]
Physical symptoms	Symptoms Checklist [41]	Post/FU between groups<br>f=0.05	 .330(-4.71, 1.58)[b]/.610(-4.80, 2.85)[b]
Pregnancy/Maternal			
Premature birth	Premature birth [37]	d=-0.88	(-1.50, -0.26)[b]
	premature birth [50]	FU2 between groups<br>0.25	 (-0.06, 0.56)[b]
	Incidence of Premature birth [35]	h=0.10	.374[b]
	Premature birth [47]	d=-0.28	.079(-0.59, 0.03)[b]
	Premature birth [52]	d=-0.01	(-1.15, 1.13)[b]
	Premature birth [39]	d=-0.24	(-0.79, 0.32)[b]

	Rate of premature birth [54]	Between groups<br>OR=.80	 .260(0.50, 1.20)[b]
	Rate of premature birth [42]	d=0.12	.641(-0.39, 0.63)[b]
Delivery method	Assisted vaginal delivery [47]	d=0.22	(-0.25, 0.70)[b]
	Delivery method [47]<br>Caesarean delivery<br>Caesarean in labor (not planned)	 d=-0.32<br>d=-0.27	 (-0.74, 0.10)[b]<br>(-0.75, 0.21)[b]
	Incidence of Cesarean section [35]	h=0.05	.640[b]
	Cesarean delivery [39]	d=-0.02	(-0.53, 0.50)[b]
Hypertension-related	Eclampsia or pre-eclampsia [47]<br>Gestational hypertension	NA<br>d=-0.28	NA[d]<br>(-1.23, 0.68)[b]
	Preeclampsia/other complications [39]	d=-0.23	(-0.79, 0.32)[b]
	First morning urine sample albumin to-creatinine ratio [52]	f=0.05	>0.05[b]
Polyhydramnios	Polyhydramnios [37]	d=-1.12	(-1.97, -0.27)[b]
Postpartum hemorrhage	PP hemorrhage [37]	d=-1.07	(-1.68, -0.46)[b]
	Incidence of PP hemorrhage [35]	h=0.10	.599[b]
Premature rupture of membranes	Premature rupture of membranes [47]	d=-0.17	(-0.66, 0.32)[b]
	Incidence of Premature rupture of membranes [35]	h=0.08	.484[b]
Maternal adverse events	Incidence of maternal adverse events [38]	OR=0.14	(0.05, 0.39)[a]
Insulin treatment	Number of Insulin-treated women [52]<br>Total contact per Insulin-treated women (total hours)	d=0.49<br>d=1.41	(-0.05, 1.04)[b]<br>(0.96, 1.85)[a]
Neonatal			
Intrauterine distress	Intrauterine distress [37]	d=-0.95	(-1.64, -0.26)[b]
Admission to NICU	Incidence of Admission to NICU [35]	h=0.22	.064[b]
	NICU admission [39]	d=0.03	(-0.63, 0.70)[b]
Malformation	Incidence of malformation [35]	h=0.07	.534[b]
Macrosomia	Macrosomia [37]	d=-0.32	(-1.09, 0.44)[b]
	Incidence of macrosomia [35]	h=0.09	.472[b]
Respiratory distress	Incidence of Neonatal asphyxia [35]	h=0.00	.973[b]
	Neonatal respiratory distress [37]	d=-0.79	(-1.37, -0.21)[b]
	Neonatal respiratory distress syndrome [54]	Between groups<br>OR=.30	 .028 (0.10, 0.90)[a]
Apgar scores	Apgar scores [38]	NA	<.05[a]
Complications	Number of neonatal complications [38]	OR=0.17	(0.06, 0.47)[a]
Mortality	Stillbirth [54]<br>Perinatal mortality	NA<br>NA	NA[d]<br>NA[d]
Birth weight	Incidence of Low birth weight [35]	h=0.23	.072[b]
	Number of low birth weight [39]	d=0.26	(-0.15, 0.67)[a]
	Birth weight [38]	NA	<.05[a]
	Birth weight [39]	d=0.48	(0.07, 0.89)[a]
**Wellness Health Outcome**			
Health management			
Medical visits	Number of prenatal examinations [36]	Between groups<br>d=1.93	 <.001(1.57, 2.30)[a]

	Medical visits [47]	d=-1.71	<.001(-2.07, -1.35)[a]
Compliance	Compliance score [38]	NA	NA[a]
	Compliance rate of the diet standards	NA	NA[a]
Diabetes and nutrition-related knowledge	Diabetes and nutrition-related knowledge [29]	NR	.353[b]
Diabetes care	Michigan Diabetes Care Profile (DCP) [37]		
	DCP-self management attitude	d=0.39	.005[a]
	DCP-self management ability	d=0.82	<.001[a]
	DCP-diet compliance	d=1.25	.000[a]
	DCP-exercise problems	d=0.35	.034[a]
	DCP-blood glucose or urine glucose monitoring problems	d=1.02	<.001[a]
Physical activity	Total accumulated MET-minutes [33]	d=0.21	≥.01[b]
	Total accumulated sedentary PA	d=0.37	≥.01[b]
	Total accumulated light PA	d=0.18	≥.01[b]
	MVPA 10-min bouts	d=0.35	≥.01[b]
	MVPA 20-min bouts	d=0.41	<.01[a]
	MVPA 30-min bouts	d=0.47	<.01[a]
Body weight gain	28 weeks gestation: % exceeding the upper limit of guidelines for total GWG [32]	RR=1.09	.12(0.98, 1.20)[b]
	32weeks gestation till delivery: % exceeding the upper limit of weekly GWG rate	RR=1.00	.90(0.94, 1.07)[b]
	32weeks gestation till delivery: Total GWG	RR=0.10	.78(-0.58, 0.77)[b]
	Body weight gain [52]	d=-0.14	(-0.54, 0.26)[b]
	At 34-36weeks gestation [33]		
	Total GWG	d=0.45	≥.05[b]
	% gained of total IOM recommendation	d=0.49	≥.05[b]
	Adherence to IOM GWG recommendations	d=-0.40	(-0.46, -0.34)[a]
	Weight change [39]	NA	NA[b]
	BMI change	NA	NA[a]
Health risk behavior			
Addiction	Alcohol use [30]	Between groups, only post time	
	Psychiatric status	d=0.36	≥.05[b]
	Drug use	d=0.22	≥.05[b]
		d=0.05	≥.05[b]
	Any drug or alcohol use [34]	OR=0.16	.020(0.04, 0.74)[a]
	4-week continuous abstinence rates of smoking [44]	OR=1.50	.220 (0.80, 2.90)[b]
Unsafe sex	Condomless vaginal sex [34]	Time·group/Between groups	
		NA/OR=5.50	NA[d]/.13(0.59, 51.21)[b]
Insomnia	Insomnia symptom severity Index [31]	Post/FU between groups	
	Sleep efficiency	d=-1.03/ d=-0.54	<.001(-0.48, -0.23)[a]/.002(-0.22, -0.05)[a]
	Nightly sleep duration	d=-0.51	.001(0.39,1 .14)[a]
	Pittsburgh sleep quality index	d=0.16	.070(-0.002, 0.67)[b]
	Insomnia caseness	d=1.04/ d=-0.34	<.001(-0.37, -0.21)[a]/.006 (-0.13, -0.02)[a]
		NA / NA	<.001(-0.35, -0.12)[d]/<.001(-0.18, -0.06)[d]
	Insomnia Severity Index [53]	g=0.44	.170(-1.25, 2.12)[b]

The decimal places of the p-value were basically written up to the 3rd place. However, the p-value with only two decimal places was the value suggested by the original author and could not be calculated in this study.

[a]= Benefit
[b]= No effect
[c]= Not report (NR)
[d]= Not applicable (NA)
[e]= Morris d
d= Cohen's d
f= Cohen's f
g= Hedges' g
h= Cohen's h
BP: blood pressure
CAR: cortisol awakening reaction
GWG: Gestational weight gain
HbA1c: hemoglobin A1c
IOM: Institute of Medicine
MVPA: moderate-to-vigorous physical activity
NICU: Neonatal Intensive Care Unit
PA: physical activity
PP: Postpartum
RBC: red blood cell
